# Circulating CD34 Positive Cells and Immunological Responses in Extremely Preterm Infants

**DOI:** 10.2147/JBM.S541742

**Published:** 2025-10-22

**Authors:** Ulrika Sjöbom, Helena Barreto Henriksson, Anders K Nilsson, Pia Lundgren, Karin Sävman, Ann Knattan Hellström, Sofia Frändberg

**Affiliations:** 1 Learning and Leadership for Health Care Professionals, Institute of Health and Care Science, Sahlgrenska Academy, University of Gothenburg, Gothenburg, Sweden; 2 The Sahlgrenska Centre for Pediatric Ophthalmology Research, Department of Clinical Neuroscience, Institute of Neuroscience and Physiology, Sahlgrenska Academy, University of Gothenburg, Gothenburg, Sweden; 3 Department of Research, Development, Education and Innovation, Sahlgrenska University Hospital, Gothenburg, Region Västra Götaland, Sweden; 4 Department of Clinical Immunology and Transfusion Medicine, Sahlgrenska University Hospital, Gothenburg, Region Västra Götaland, Sweden; 5 Department of Ophthalmology, Sahlgrenska University Hospital, Gothenburg, Region Västra Götaland, Sweden; 6 Department of Pediatrics, Institute of Clinical Sciences, Sahlgrenska Academy, University of Gothenburg, Gothenburg, Sweden; 7 Department of Neonatology, the Queen Silvia Children’s Hospital, Sahlgrenska University Hospital, Gothenburg, Region Västra Götaland, Sweden

**Keywords:** CD34, CD45, hematopoietic stem cells, immunological response, infection, preterm infants

## Abstract

**Background:**

The dynamic changes of the hematopoietic system during fetal development may be disrupted by preterm birth. Hematopoietic stem and progenitor cell (CD34^+^) levels are poorly investigated in preterm infants, particularly in relation to immune responses and morbidities. This is partly because of low blood volumes, which raise ethical concerns and limit specific sampling for research studies. To overcome this problem, we used residual blood from routine clinical testing to monitor CD34^+^ cell counts in the first months of life. Our aim was to characterize the dynamics of circulating CD34^+^ cells and explore associations with prenatal and postnatal clinical events.

**Methods:**

We retrieved residual blood samples from nine infants born <28 weeks gestational age (GA), collected from birth through eight postnatal weeks. CD34^+^ cell count was assessed using flow cytometry. The number of nucleated red and white blood cells, and hemoglobin concentration were also measured.

**Results:**

Median (min-max) GA was 25+0 (22+3─27+5) weeks. CD34^+^ cell counts at birth ranged from 19 to 284 x 10^6^ cells/L. Between days 0 and 1, CD34^+^ cell count increased in four infants and decreased in four. By day 7, the proportion of CD34^+^ of total nucleated blood cells was significantly lower than at birth (p=0.018). High inter- and intra-individual variability in CD34^+^ cell count was observed. Notably, the highest CD34^+^ cell levels coincided with maternal or infant infections.

**Conclusion:**

This pilot study demonstrates the feasibility of longitudinal monitoring of CD34^+^ hematopoietic stem and progenitor cells in extremely preterm infants using residual clinical blood samples. While limited by a small sample size, the study provides preliminary insights into early immune function and highlights directions for future research in larger cohorts.

## Introduction

Globally, preterm birth is the leading cause of neonatal morbidity and mortality.^1^ Organs undergo continuous maturation from fetal to extrauterine life. Preterm birth interrupts this physiological development, potentially leading to organ damage or dysfunction. Several neonatal morbidities and postnatal complications, including intraventricular hemorrhage (IVH), bronchopulmonary dysplasia (BPD), retinopathy of prematurity (ROP) and sepsis, are strongly associated with immaturity.^2^

The hematopoietic system shows dynamic changes during fetal development,^3^,^4^ with stem and progenitor cells in the peripheral blood playing a key role in hematopoietic cell renewal during embryogenesis. These cells migrate from extramedullary stem cell sources through the peripheral circulation to colonize bone marrow, where definitive hematopoiesis is established.^5^ Hematopoietic stem and progenitor cells express the highly glycosylated transmembrane surface protein CD34 from the early stages of development. CD34, in conjunction with the leucocyte marker CD45, is typically used to identify and isolate hematopoietic stem and progenitor cells (CD34^+^ cells) in clinical laboratories.^6^,^7^

Several factors have been proposed to influence the number of CD34^+^ cells in cord blood, including infant birth weight, the length of labor, the way of delivery, changes in oxygen saturation after initial breathing, the exocrine role of the placenta, and blood collection methods.^8–12^ Higher proportions of CD34^+^ cells in cord blood have been reported in preterm infants compared to term infants,^13^ and lower gestational age (GA) has been related to higher CD34^+^ cord blood cell count.^14^ In both term and preterm (<37 weeks GA) infants, CD34^+^ cell counts in peripheral blood decline after birth.^8^,^15^ A high number of hematopoietic stem cells in preterm infant cord blood has been associated with a reduced risk of prematurity-related morbidities.^13^ The investigation of CD34^+^ hematopoietic stem and progenitor cells in extremely preterm infants is of particular relevance, as these cells contribute to hematopoiesis, stem cell migration, and immune maturation.^5^ In neonates, hematopoietic stem cells are highly proliferative with a strong capacity for self-renewal.^16^,^17^ Understanding their postnatal behavior may provide important prognostic information regarding susceptibility to infection and other morbidities of prematurity and could inform targeted interventions to improve neonatal outcomes.

The development of circulating CD34^+^ cells in extremely preterm infants (<28 weeks GA) remains poorly described. In all studies involving blood sampling of preterm infants, the negative effects associated with frequent blood sampling and the consequent risk of iatrogenic anemia must be considered. Additionally, while evaluating hematopoietic factors, the risk of bias from frequent blood sampling and transfusions of blood components must be considered. The estimated blood volume of a preterm infant is approximately 70 mL/kg body weight,^18^,^19^ and cumulative sampling can exceed 50% of this volume within the first two weeks of life.^20^ Such losses are associated with an increased need for red blood cell transfusions^21^,^22^ and a higher risk of preterm morbidities.^20^ The EPITOP study is a feasibility study designed to evaluate the use of residual clinical blood samples for biomarker research while minimizing clinical risks.^23^ As a pilot within this framework, the present study aimed to assess the feasibility of using salvaged blood volumes to longitudinally evaluate postnatal changes in circulating CD34^+^ hematopoietic stem cells in extremely preterm infants over the first eight weeks of life and to explore the influence of pre- and postnatal events on CD34^+^ cell levels.

## Materials and Methods

### Infant Characteristics and Data Collection

This feasibility study was approved by the Swedish Ethical Review Authority (reference no 2019–03110) and complies with the Declaration of Helsinki.^24^ Ten infants born <28 weeks GA at the Queen Silvia Children’s Hospital, Gothenburg, Sweden, between September 2020 and June 2021 were included after parental informed consent.

Infant birth characteristics, including information on suspected maternal infection and the onset of delivery, were retrospectively recorded. Postnatal events such as blood transfusions, sepsis (with or without positive cultures), the number of laboratory tests, and preterm morbidities were also documented. Preterm morbidities were defined as follows:

- Retinopathy of prematurity (ROP): diagnosed according to the International Classification of Retinopathy of Prematurity^25^
- Intraventricular hemorrhage (IVH): diagnosed according to modified Papile criteria^26^,^27^
- Bronchopulmonary dysplasia (BPD): defined as the need for supplemental oxygen at 36 weeks postmenstrual age
- Necrotizing enterocolitis (NEC): Bell’s stages 2–3^28^
- Patent ductus arteriosus (PDA): requiring medical or surgical treatment

Laboratory data were extracted from medical records, including levels of C-reactive protein (CRP; Alinity c CRP Vario Reagent Kit, Abbott), interleukin-6 (IL-6; Elecsys ECLIA kit, Cobas, Roche Diagnostics), and glucose concentrations obtained from blood gas analyses (Siemens RAPIDpoint 500/500e).

### Sample Collection

Cell counts were determined in heparinized residual blood from blood-gas syringes. Following the blood gas analysis, whole blood samples were stored and transported at room temperature, with a maximum storage time of three days prior to analysis. Blood cell counts were analyzed every other day during the infants’ first two weeks of life, twice weekly during postnatal weeks 3–4, and once weekly during postnatal weeks 5–8. If the first sample was collected on the day of birth, it was recorded as postnatal day 0.

### Blood Cell Count

Samples were analyzed for white blood cell (WBC) count, nucleated red blood cell (NRBC) count, and hemoglobin (Hb) concentration using a CELL-DYN Sapphire hematology analyzer (Abbott, Lake Bluff, USA). All samples were measured at a 1:10 dilution.

Sample stability over time was evaluated over a three-day period. The intermediate series coefficient of variation (CV) was <0.1% for Hb and 11.2% for nucleated blood cells (NBC), defined as the sum WBC and NRBC counts.

### Cell Surface Marker Profiling

Cell profiling of hematopoietic stem cells was performed using flow cytometry targeting CD34 and CD45,^29^ with BD TruCount vials (BD Biosciences, 7 Franklin Lakes, USA). The antibodies used were CD45-FITC-A and CD34-PE-A (BD Biosciences). Cell viability was assessed using 7-AAD PerCP-Cy5-5-A solution, following standard protocols provided by the manufacturer (BD Biosciences). For each sample, 100,000 cells were analyzed using a FACS Canto II flow cytometer (BD Bioscience). Flow cytometric gating was performed according to the International Society of Hematotherapy and Graft Engineering (ISHAGE) guidelines,^7^ and a representative gating strategy is shown in Figure S1.

Sample stability for CD34^+^ cells over a three-day period was assessed by evaluating cell viability (>98%) and calculating the intermediate series CV, which was 8%. No correlation was observed between the time from blood draw to analysis and the number of CD34^+^ cells.

### Statistics

Due to the limited number of cases and skewed data, median with range (min-max) and interquartile range (q1-q3) were employed to report clinical data, including concentrations of CD34^+^ cells and percentages of CD34^+^ cells relative to total NBC. Longitudinal changes were analyzed using the Wilcoxon signed-rank test, with results reported as median and 95% confidence intervals (CI). Associations with demographic variables were assessed using Spearman’s rank correlation. P-values below 0.05% were considered significant.

Statistical analyses were performed using IBM SPSS Statistics (Version 29.0.0.0, IBM, Armonk, NY) and visualized in R (version 4.3.0; The R Foundation for Statistical Computing) using the ggpubr and ggplot2 packages. The study follows the Strengthening the Reporting of Observational Studies in Epidemiology (STROBE) reporting guidelines.

## Results

Ten extremely preterm infants were initially enrolled in the study. One infant was excluded because blood samples were received outside the acceptable time frame for analysis. The median GA (range [q1-q3]) of the included infants was 25+0 weeks (22+3 to 27+5 weeks, [23+2 to 26+1 weeks]), and the median birth weight was 745 grams (470 to 1240g [625 to 987.5g]). Of the nine included infants, six were female, and three were male. Four infants were delivered vaginally.

One infant died during the first postnatal week, four developed severe ROP, three had any stage of IVH, five developed BPD, and three were treated for PDA. No cases of NEC were observed.

A total of 85 blood samples were analyzed for CD34^+^ cell count. One sample with a cell viability of 45% was excluded. The viability of the CD34^+^ cells in the remaining samples ranged from 84.1% to 99.8%.

### Birth Levels of CD34^+^ Cell Count and Maternal Infection

On day 0, CD34^+^ cell counts had a median (range [q1-q3]) of 49.7×10^6^ cells/L (19 to 284×10^6^ cells/L [21 to 200×10^6^ cells/L]), with the highest level observed in an infant born to a mother with verified group B streptococcal urinary tract infection (Table 1). No significant correlations were found between CD34^+^ cell count on day 0 and GA (rho=0.06, p=0.888) or birth weight (rho=−0.07, p=0.882).

**Table 1:** Description of the Nine Included Infants and CD34^+^ Cell Count Variables

Subject	1	2	3	4	5	6	7	8	9
GA week	23	26	22	23	24	26	25	25	27
Suspected maternal infection	Yes	Yes	Yes	Yes	No	No	No	No	No
Reason for preterm birth	PPROM, mother GBS	PPROM	Preterm labor	PPROM	PPROM	Preterm labor	Pathological CTG	Pathological CTG	Pathological CTG
Sepsis, postnatal day (culture ±)	No	No	0 (-)	9 (+) and 27 (+)	9 (+)	No (day 14 *S. aureus* infection)	31 (+)	7 (-) and 20 (-)	No
Number of samples (first/last day for sample)	12 (0/50)	6 (0/15)	2 (1/3)	14 (0/51)	11 (0/31)	9 (0/29)	13 (0/53)	15 (0/47)	4 (0/6)
CD34^+^ count at day 0/1[10^6^ cells/L]	284/351	226/217	NA/88	28/49	19/167	21/69	22/17	72/21	120/98
Postnatal day at maximum CD34^+^ count and cell number [10^6^ cells/L]	1 (351)	0 (226)	1 (88)	11 (304)	1 (167)	1 (69)	53 (67)	13 (74)	0 (120)
Postnatal day at minimum CD34^+^ count and cell number	18 (10)	15 (7)	3 (21)	9 (20)	20 (11)	0 (21)	26 (11)	27 (12)	6 (32)

**Abbreviations**: GA; gestational age, GBS, group B streptococcus; CTG, cardiotocography; NA; not analyzed, PPROM, preterm premature rupture of membranes.

### Postnatal Patterns of CD34^+^ Cell Count

**Figure 1:**
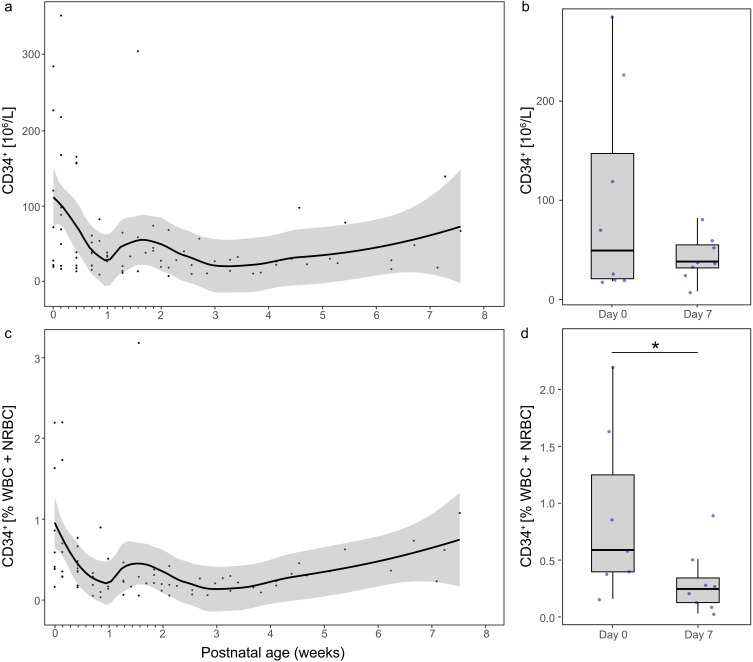
Longitudinal CD34^+^ cell counts in blood from the first day of life through postnatal week 8 (n*=*9). (**a**) and (**b**) show CD34^+^ cell counts per blood volume, while (**c**) and (**d**) show CD34^+^ cells as a percentage of total nucleated blood cells (white blood cells [WBC] + nucleated red blood cells [NRBC]). In (**a**) and (**c**), individual measurements are represented as dots, with trend lines estimated using locally estimated scatterplot smoothing (LOESS); shaded areas representing the 95% confidence interval. In (**b**) and (**d**), boxplots display medians (horizontal line), first and third quartiles (box), and whiskers extending to 1.5 x the interquartile range. *p<0.05.

Longitudinal levels of CD34^+^ cells expressed per blood volume and normalized to total nucleated cells (NBC) are illustrated in Figure 1. Eight infants had CD34^+^ cell data available on both day 0 and day 1, and seven infants had samples collected beyond 14 days of life. Between day 0 and day 1, four infants showed an increase in CD34^+^ cell count, while four showed a decrease. The largest individual increase between day 0 and day 1 was from 19 to 167×10^6^ cells/L ─ an 8-fold rise. The largest individual decrease between day 0 and day 1 was from 72 to 21×10^6^ cells/L.

The proportion of CD34^+^ cells relative to total NBC was significantly higher on day 0 compared to day 7 (median [95% CI] 0.6% [0.4–2.2%] vs 0.2% [0.1–0.5%], p=0.018, n*=*7, Figure 1d). However, the absolute CD34^+^ cell count did not significantly differ between these time points (median [95% CI] 49.7×10^6^ cells/L [21.0–226.0×10^6^ cells/L] versus 36.4×10^6^ cells/L [26.3–53.6×10^6^ cells/L], p=0.327, n*=*8, Figure 1b).

### Individual CD34^+^ Cell Count in Relation to Inflammation-Related Biological Parameters

**Figure 2:**
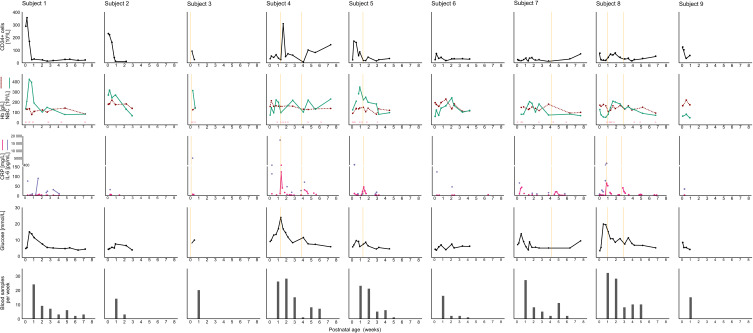
Individual trajectories of blood CD34^+^ cell counts and inflammation-related parameters. The top panel shows CD34^+^ cell count per blood volume. The second panel displays hemoglobin (Hb) levels, and nucleated blood cell (NBC) count, defined as white blood cells + nucleated red blood cells. The third panel presents serum concentrations of the inflammatory markers interleukin-6 (IL-6) and C-reactive protein (CRP). The fourth panel shows serum glucose concentrations. The bottom panel illustrates the number of blood samples collected per week. Vertical Orange lines indicate episodes of sepsis. Red blood transfusions are marked with pink dots at the bottom of the second panel.

To explore the relationship between postnatal CD34^+^ cell counts, immune responses, and related hematological parameters, individual trajectories of CD34^+^ cells were plotted alongside Hb, NBC, IL-6, CRP, sepsis, blood glucose levels, and the frequency of iatrogenic blood sampling (Figure 2).

In some infants, such as subjects 4 and 8, elevated levels of inflammatory markers (CRP and IL-6) and sepsis coincided with increased circulating CD34^+^ cell counts. In subject 5, CD34^+^ cell counts rose immediately after birth, concurrent with a high IL-6 concentration. A second, smaller increase in both CD34^+^ cells and CRP was observed during a sepsis episode in the second postnatal week. For subject 7, no samples were available in the week following sepsis.

Overall, no consistent pattern was observed between CD34^+^ cell counts, inflammatory markers, or other blood parameters across individuals. During the first week of life, an average of 22 blood samples were taken from the infants, and they received an average of 2.5 red blood cell transfusions during the first two weeks.

## Discussion

This feasibility study assessed longitudinal CD34^+^ cell levels in residual peripheral blood samples from extremely preterm infants and explored their associations with clinical variables and blood parameters. CD34^+^ cell counts exhibited marked intra- and interindividual variability over time. The highest levels were observed in association with maternal or neonatal infections, although the small sample size limited the possibility of formal statistical analysis.

Recognizing the potential negative effects of studies involving frequent blood sampling on preterm infants is crucial. In preterm infants, several factors, including frequent blood sampling, immediate cord clamping, and transfusions of adult red blood cells, have been associated with reduced levels of fetal hemoglobin.^20^,^30^ These interventions may also affect the number of hematopoietic stem cells in peripheral blood, as suggested by increased hematopoietic stem cells following delayed cord clamping.^31^ In our study, most infants received adult red blood cell transfusion, and all underwent frequent blood sampling during the first week of life. The amount of blood loss, presence of anemia, reduced levels of fetal hemoglobin, and the number of blood transfusions have been associated with increased risk for several neonatal morbidities affecting especially preterm infants, such as ROP,^32–34^ BPD,^20^,^35^ and NEC,^36^,^37^ and also impaired neurodevelopment.^34^ Sepsis^38^ and other prematurity-related morbidities predispose infants to additional complications of prematurity, including BPD.^39^ These interrelationships highlight that prematurity-related morbidities are not independent events, and such associations need to be considered in statistical analyses to avoid confounding and to ensure accurate interpretation of results.

In preterm infants, circulating CD34^+^ cell levels have been associated with various neonatal morbidities.^40–44^ Higher levels of hematopoietic stem cells have been reported in peripheral blood from preterm infants with brain injury^42^ and lung disease,^40^ and in cord blood hematopoietic stem cells have been associated with the development of several premature birth complications.^13^,^45^ However, the small sample size in our study precluded a meaningful analysis of associations between CD34^+^ cells and clinical outcomes.

Our study demonstrated an overall decrease in CD34^+^ cell count during the first postnatal week, with substantial inter-individual variability. Li et al^14^ reported an increase in CD34^+^ cell counts in preterm infants within 2–8 hours after birth, whereas term infants showed a decrease during the same period. However, CD34^+^ cell levels declined in both groups after 8 hours. Similarly, Gonzales et al^8^ observed a decrease in peripheral blood CD34^+^ levels between 3 and 60 hours after birth in both preterm and term infants. Notably, their cohort included infants born to mothers colonized with group B streptococcus who received intrapartum antimicrobial prophylaxis, similar to the infant in our study with the highest observed CD34^+^ level.

Tong Leung et al^46^ reported lower circulating CD34^+^ cell levels at the time of sepsis, followed by increased levels one week later. In our study, a few infants had samples available both shortly before and after sepsis diagnosis. Notably, in one infant (subject 4), a rapid rise and subsequent decline in CD34^+^ cell count was observed in close temporal association with sepsis. Similarly, Das et al^47^ described dynamic changes in the blood immune profile during neonatal sepsis and identified plasma amphiregulin as a sepsis-associated inflammatory marker. CD34^+^ hematopoietic stem cells were among the cell types with the highest levels of amphiregulin expression.^47^ In adults, circulating CD34^+^ cell levels have also been shown to increase during the first week following sepsis.^48^ Collectively, these findings, along with our results, suggest that CD34^+^ cell counts may fluctuate in response to bacterial infections in preterm infants. Inflammatory stimuli and pro-inflammatory cytokines can drive mobilization, proliferation, and release of these cells from the bone marrow into circulation.^5^,^49^ This interplay between infection-driven inflammation and CD34^+^ cell dynamics may therefore be an important determinant of immune development and outcomes in extremely preterm infants.

One of the strengths of this study is the use of residual blood from routine clinical sampling, allowing for high-resolution longitudinal data without subjecting infants to additional blood loss. However, this sampling strategy introduces a potential bias, as more samples are collected from critically ill infants. Despite the relatively high sampling frequency, not all dynamic changes were captured, and higher temporal resolution may be needed in future studies. We analyzed CD34^+^/CD45^+^ cells, which is consistent with a hematopoietic stem and progenitor cell phenotype and excludes non-hematopoietic CD34^+^ populations such as endothelial cells.^6^,^7^ However, using only these two markers does not allow discrimination between long-term hematopoietic stem cells, short-term stem cells, and more lineage-committed progenitors. Additional markers (eg CD38, Lin, CD10, CD7 and CD45RA) would be required to resolve these subpopulations and provide a more detailed characterization.^50^ The small sample size remains a major limitation, as it also precludes adequate adjustment for potential confounding factors such as blood transfusions, frequent sampling, and comorbidities. Nevertheless, our findings demonstrate the feasibility of using residual clinical samples to monitor CD34^+^ cell levels and underline the potential of this approach for future studies. Larger cohorts will be needed to more robustly investigate associations between CD34^+^ cell dynamics, infection, and prematurity-related morbidities, as well as to evaluate the impact of interventions and clinical factors such as blood transfusion, timing of cord clamping, and sampling frequency.

Importantly, these preliminary results suggest that CD34^+^ cell dynamics may reflect aspects of immune competence in extremely preterm infants, and future studies may clarify whether such measurements could serve as prognostic biomarkers or inform therapeutic strategies aimed at improving outcomes in this vulnerable population. Moreover, our study indicates that samples taken over shorter intervals may be required to fully capture the rapid and dynamic changes in CD34^+^ cells during the early postnatal period.

## Conclusion

This exploratory pilot study demonstrates the feasibility of using residual clinical blood samples to longitudinally monitor CD34^+^ hematopoietic stem and progenitor cells in extremely preterm infants. We observed dynamic postnatal changes in CD34^+^ cell levels, and our findings suggest that the ability of preterm infants to mobilize these cells in response to clinical stress may provide insights into early immune function. However, the small sample size and exploratory design represent important limitations that restrict generalizability and preclude definitive conclusions. Despite these constraints, the study adds to the limited knowledge in this understudied field and highlights avenues for future research in larger cohorts, which may ultimately inform prognostic or therapeutic strategies for this vulnerable population.

## Data Availability

The datasets generated and/or analyzed during the current study are not publicly available due to ethical permits and The General Data Protection Regulation (GDPR) Regulation (EU) 2016/679 on the protection of natural persons with regard to the processing of personal data and on the free movement of such data law regulates the availability of personal data, but deidentified data are available from the corresponding author on reasonable request.

## Abbreviations

BPD, bronchopulmonary dysplasia; CRP, C-reactive protein, GA, gestational age; IL-6, interleukin 6; IVH, intraventricular hemorrhage; NBC, nucleated blood cells, NEC, necrotizing enterocolitis; NRBC, nucleated red blood cells, PDA, patent ductus arteriosus, ROP, retinopathy of prematurity.
